# Correction: Particle swarm optimization algorithm-based PI inverter controller for a grid-connected PV system

**DOI:** 10.1371/journal.pone.0259358

**Published:** 2021-10-26

**Authors:** M. F. Roslan, A. Q. Al-Shetwi, M. A. Hannan, P. J. Ker, A. W. M. Zuhdi

The following information is missing from the Acknowledgments: This work was funded by the Ministry of Higher Education Malaysia under Long-Term Research Grant Scheme (LRGS) Program Project Grant No. 20190101LRGS Through Universiti Tenaga Nasional. The Authors also Gratefully Acknowledge the Bold Refresh Publication Fund 2021 (J5100D4103) for the Continuous Support in Publishing the Research.
