# Supplementary material for: Particle swarm optimization algorithm-based PI inverter controller for a grid-connected PV system
Source: PLoS One. 2020 Dec 23;15(12):e0243581. doi: 10.1371/journal.pone.0243581 (PMC7757891; doi:10.1371/journal.pone.0243581)
Supplement: Attachment — Submitted filename: Response to reviewers.docx [file pone.0243581.s001.docx]

**Response to Reviewers**

**Dear Editor-in-Chief, *October 12,2020***

*PLOS ONE*

**Revised Submission of Manuscript Title as “*Particle Swarm Optimization Algorithm-based PI Inverter Controller for a Grid-connected PV System*”, manuscript ID: *PONE-D-20-26206.***

Authors: M.F.Roslan*, Ali. Q. Al-Shetwi, M.A. Hannan, P. J. Ker, A. W. M. Zuhdi.

The above matter is referred.

We are submitting the revision herewith to the *PLOS ONE* including a copy of the response to reviewers, edited manuscript with track changes, and clean edited manuscript without the track changes.

**Dear Professor,**

Thank you for your and reviewers’ constructive remarks, comments and suggestions on our manuscript which helped us to further improve the quality of the revised manuscript. We have carefully revised the manuscript based on the reviewer’s comments and suggestions. We hope that the revision has improved the manuscript to a level of satisfaction. All the comments and suggestions raised by the reviewers are addressed and the corrections are highlighted in red font in the edited manuscript with track changes. Our responses are given in a point-by-point manner in the next pages.

Amended funding statement for this work: “This work was funded by the Ministry of Higher Education, Malaysia under Universiti Tenaga Nasional using grant no. 20190101LRGS.”

Thank you,

M F Roslan

Department of Electrical Power Engineering

College of Engineering, Universiti Tenaga Nasional

43000 Kajang, Selangor Darul Ehsan, Malaysia

E-mail: Mfirdaus@uniten.edu.my

**Reviewer # 1 Responses**

**Comments:**

Reviewer #1: Authors proposed the performance of a control strategy for an inverter in a three-phase grid-connected PV system. There are some concerns, as listed below.

1) The authors are recommended to highlight the novelty and contribution of the work.

**Authors Response:** Thank you for your recommendation. According to the suggestion, we have highlighted the significant novelty and contribution of the work. Please find the red highlighted font on page 5 and 6 in the edited manuscript.

**Actual Changes Implemented:**

The implementation of PSO algorithm into the PI controllers which provide an optimized controller to improve the power quality performance of the inverter systems. Therefore, the contributions and key highlights of the paper are:

i. It proposes an optimized controller-based PSO algorithm to obtain the optimum values of $K_{p}$ and $K_{i}$ in real-time operation to improve the power quality and stability of the three-phase grid-connected PV inverter system.
ii. It solves the complicated constraints of the optimization problem, while obtaining an optimum value of the PI controllers’ parameters by reducing transient response, minimising time overshoot, and obtaining low steady-state error due to variation in loads conditions.
iii. It offers a real-time operation of the PV inverter system with optimized controller by minimizing the error as much as possible, thus finding the best optimum parameters of PI controllers under load variation condition.

2)The authors are recommended to highlight the advantages of their proposed method in comparison with conventional methods.

**Authors Response:** Thank you for your valuable suggestion. The paragraph explaining the advantages of the proposed method in comparison with conventional methods are added at page 17 and 18 in the edited manuscript.

**Actual Changes Implemented:**

The main advantages and disadvantages of the conventional Ziegler-Nichols technique, fuzzy logic controller, neural fuzzy logic, GA, and PSO algorithm are described in Table 3. From the table, we can see that the PSO algorithm has a simple set of parameters during implementation, which requires a shorter time to converge to an optimum solution compared to other techniques. Furthermore, the PSO algorithm does not require inferences rules during implementation compared to the fuzzy logic controller and neural fuzzy logic, which requires a time-consuming trial and error process. Therefore, the PSO algorithm leads to a simple and fast implementation process with single solution space to obtain the optimum values of the PI controller parameters with shorter time responses.

Table 1: Advantage and disadvantage of control algorithm

| **Algorithm** | **Data Training** | **Rules** | **Function evaluation** | **Parameter setting** | **References** |
| --- | --- | --- | --- | --- | --- |
| Ziegler-Nichols | No | No | No | Simple with calculation | [6] |
| Fuzzy Logic controller | No | Yes | No | Simple but trial and error | [7] |
| Neural Fuzzy Logic | Yes | Yes | No | Huge set of parameters | [9],[11] |
| GA | No | No | Yes | Huge set of parameters | [12] |
| PSO | No | No | Yes | Simple set of parameters | [17],[18] |

3) How the proposed method has improved the performance evaluation compared with JAYA algorithm [1]?

[1] Wang L, Huang C, Huang L. Parameter estimation of the soil water retention curve model with Jaya algorithm[J]. Computers and Electronics in Agriculture, 2018, 151: 349-353.

**Authors Response:** Thank you for your sharp observation. The performance evaluation of the proposed method is compared and justified with the suggested method which is JAYA Algorithm. We would gladly appreciate if the reviewer accepted our justification. Please find the red highlighted font on page 4 in the edited manuscript. We would gladly appreciate if the reviewer can accept our justification. Thank you.

**Actual Changes Implemented:**

The Jaya algorithm optimization is also introduced to tune the PID controller for minimizing frequency deviation in hybrid electric vehicle (HEV) applications [14]. One study implemented the Jaya algorithm optimization in the parameter estimation of the soil water retention to validate the effectiveness of the algorithm [15]**.**

The PSO algorithm is implemented in this study is to estimate the PI controller parameters as well as optimized the performance of PI controller to improve system performance. PSO optimization algorithm is implemented in the designated model of three phase inverter grid-connected PV system to estimate and obtain an optimum values of PI controller parameters under load variation and off-grid condition. Furthermore, the power quality enhancement also achieved by minimizing the error in inverter control strategy as much as possible in real time responses. Wang et al (2018) proposed Jaya algorithm that has also good performance which has been implemented in soil water retention for parameter estimation. The Jaya algorithm proved their superiority among others algorithm such as Differential evolution (DE) algorithm and PSO algorithm in case of small amount of data estimation.

[14] A. Annamraju and S. Nandiraju, “Coordinated control of conventional power sources and PHEVs using jaya algorithm optimized PID controller for frequency control of a renewable penetrated power system,” *Prot. Control Mod. Power Syst.*, vol. 4, no. 1, 2019, doi: 10.1186/s41601-019-0144-2.

[15] L. Wang, C. Huang, and L. Huang, “Parameter estimation of the soil water retention curve model with Jaya algorithm,” *Comput. Electron. Agric.*, vol. 151, no. July 2017, pp. 349–353, 2018, doi: 10.1016/j.compag.2018.06.024.

4) Authors should include references from 2019 to 2020.

**Authors Response:** Thank you for your sharp suggestion. A few suggested references from 2019 to 2020 are included in the edited manuscript.

**Actual Changes Implemented:**

[14]	A. Annamraju and S. Nandiraju, “Coordinated control of conventional power sources and PHEVs using jaya algorithm optimized PID controller for frequency control of a renewable penetrated power system,” *Prot. Control Mod. Power Syst.*, vol. 4, no. 1, 2019, doi: 10.1186/s41601-019-0144-2.

[15]	L. Wang, C. Huang, and L. Huang, “Parameter estimation of the soil water retention curve model with Jaya algorithm,” *Comput. Electron. Agric.*, vol. 151, no. July 2017, pp. 349–353, 2018, doi: 10.1016/j.compag.2018.06.024.

[18]	S. Kakkar, R. K. Ahuja, and T. Maity, “Performance enhancement of grid-interfaced inverter using intelligent controller,” *Meas. Control (United Kingdom)*, vol. 53, no. 3–4, pp. 551–563, 2020, doi: 10.1177/0020294019879171.

[23]	A. Q. Al-Shetwi, M. A. Hannan, K. P. Jern, M. Mansur, and T. M. I. Mahlia, “Grid-connected renewable energy sources: Review of the recent integration requirements and control methods,” *J. Clean. Prod.*, vol. 253, p. 119831, 2020, doi: 10.1016/j.jclepro.2019.119831.

[24]	J. Ramos-Hernanz, I. Uriarte, J. M. Lopez-Guede, U. Fernandez-Gamiz, A. Mesanza, and E. Zulueta, “Temperature based maximum power point tracking for photovoltaic modules,” *Sci. Rep.*, vol. 10, no. 1, pp. 1–10, 2020, doi: 10.1038/s41598-020-69365-5.

[33]	N. A. Selamat, T. O. Ramih, A. R. Abdullah, and M. S. Karis, “Performance of PID Controller Tuning based on Particle Swarm Optimization and Firefly Algorithm,” *Int. J. Recent Technol. Eng.*, vol. 8, no. 3S2, pp. 225–230, 2019, doi: 10.35940/ijrte.c1042.1083s219.

[38]	M. Vinay Kumar and U. Salma, “A novel voltage regulation technique for a three phase grid connected photovoltaic system using fuzzy fractional order pi controller,” *J. Adv. Res. Dyn. Control Syst.*, vol. 11, no. 8 Special Issue, pp. 2979–2991, 2019, doi: 10.35940/ijrte.D8937.118419.

[43]	H. Ghazvinian *et al.*, “Integrated support vector regression and an improved particle swarm optimization-based model for solar radiation prediction,” *PLoS One*, vol. 14, no. 5, 2019, doi: 10.1371/journal.pone.0217634.

--------------------------------------------------------

***[Thank you for your time in reviewing our paper. The authors]***

***----------------------------------------------------------------------------------------------------------------***

**Reviewer #2 Responses**

**Comments:**

1) How to design an effective and efficient PI controller for a three-phase grid-connected inverter to obtain satisfied power quality performance is widely known research topics. Only the PI controller design for simulation model is not enough. Authors should be added a new section that is experimental section if funds allow.

**Authors Response:** Thank you for your valuable suggestion. We agreed with the reviewer that the design of PI controller only is not enough for power quality performance. We are happy to inform that we will consider the experimental section in our next paper. Please find the red highlighted font on page 33 in the edited manuscript.

**Actual Changes Implemented:**

The intermittency of output generation by the PV system based on sun irradiation leads to unstable power supply to the loads especially where utility is unavailable. Thus, improper control and optimal design of controller leads to pure of power quality and stability performance of the system. Therefore, in this study the PSO algorithm is implemented in the PI controller to obtain an optimum values of PI controller parameters to improve system performance in real time simulation system. In addition, a real time experimental setup of the system has been considered as future work of this system.

2) In a grid-connected photovoltaic system, the frequency and the harmonics of voltage is determined by the larger grid, but power quality, stability and power mismatch can be improved by the three-phase grid-connected inverter, so the author needs to do some more jobs about power quality.

**Authors Response:** Thank you for your valuable suggestion. We agreed with the reviewer that the power quality, stability and power mismatch can be improved by the three-phase grid-connected inverter system. Accordingly, we have also considered the few parameters in this study which related to the power quality enhancement in the three-phase grid-connected PV inverter system. The considered parameter is reduced transient response of DC link voltage, minimizing time overshoot, THD analysis, and obtaining a low steady-state error under load variation conditions. Please find the red highlighted font on page 5 in the edited manuscript. We would gladly appreciate if the reviewer can accept our justification. Thank you.

**Actual Changes Implemented:**

The proposed controller scheme is implemented using a synchronous reference frame with two PI controllers. The feed-forward compensation is applied to the inner current control loop of the inverter to achieve high dynamic response. In view of the above discussion, the references mentioned above have limitations in terms of insufficient process information, less robustness, longer computation time than PSO, facing parameter complexities and coding difficulties to find the best fitness value. Moreover, they did not sufficiently discuss the effects of inverter optimization to improve the power quality performance, reduce the DC link voltage fluctuation, stabilise the output current and voltage, smooth the power flow, decrease the harmonics, and stabilise the frequency in the grid-connected PV inverter. Therefore, this work aims to improve the power quality performance of the inverter system by reducing transient response, minimising time overshoot, and obtaining low steady-state error due to variation in loads as well as DC link stabilization. In addition, the PSO is used to obtain the optimum values of the PI control parameters and reduce error to enhance the voltage and frequency stability with shorter time and less complexity.

3) This manuscript does not bring any new knowledge on evolutionary algorithm, so the innovations focus on novel applications. The evolutionary algorithm, such as BCEO, PSO, should be implemented in the novel model in this manuscript, and the convergence process of PSO and BCEO should be shown in Fig.16.

**Authors Response:** Thank you for your valuable suggestion. The evolutionary algorithm such as BCEO are already implemented in the model. According to the suggestion, we have compared the convergence curve with the BCEO and PSO algorithm in Fig.16. it is observed that, PSO has superior performance compare to BCEO. Thus, we have chosen a PSO as an optimization algorithm in this study. After running the model with optimization algorithm, the result for convergence are plotted in the Fig. 16 accordingly. The convergence curve of the both algorithms are also presented in the Fig. 16. Please find the red highlighted font at page 28 in the edited manuscript, Thank you.

**Actual Changes Implemented:**

Fig. 16 shows that the PSO algorithm reduces the fitness function and converges the value at 45 iterations to reach the best result as compared to the Binary Coded Extremal Optimization (BCEO) algorithm, which converges at 67 iterations. The optimization has been run for 100 iterations to determine the best values of the PI controller parameters $K_{p}$ and $K_{i}$ obtained by using the PSO algorithm.

**Fig 16. Relationship curve between fitness function and iteration.**

--------------------------------------------------------

***[Thank you for your time in reviewing our paper. The authors]***

***----------------------------------------------------------------------------------------------------------------***
